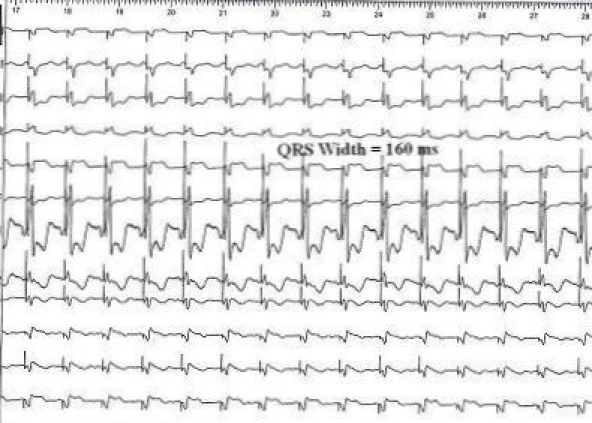# Diffuse Fragmented QRS as an Index of Extensive Myocardial Scar

**Published:** 2010-01-07

**Authors:** Amir Aslani, Amir Tavoosi, Zahra Emkanjoo

**Affiliations:** Pacemaker and Electrophysiology Department, Rajaei Cardiovascular Medical and Research Center, Iran University of Medical Sciences

**Keywords:** Fragmented QRS, Myocardial Scar

## ECG Presentation

A 70-year-old man with history of previous myocardial infarction was referred for cardiac resynchronization therapy (CRT) implantation. Echocardiography showed global wall motion abnormality and non-viable myocardium with ejection fraction of 15% and large apical aneurysm. Electrocardiography (ECG) revealed sinus rhythm with wide QRS (200 ms). Of note is the finding of marked fragmentation of QRS in limb leads and V4 - V6.

**Figure 1A:**
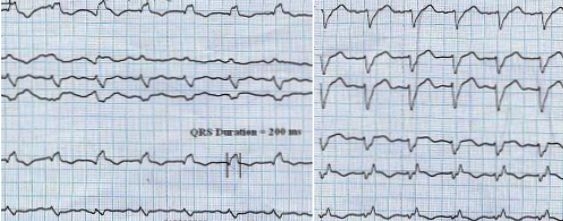


This may be an index of extensive myocardial scar (Figure 1A). CRT was implanted for the patient. ECG taken immediately after CRT implantation, showed that QRS width was decreased to 160 ms (Figure 1B).  As noted in other studies, reduction in QRS duration just after CRT implantation is an indirect sign of response to CRT in heart failure patients.

Previous studies suggest that the regional myocardial scar is associated with alteration in QRS morphology, leading to terminal conduction delay or a fragmentation of the QRS complexes on 12-lead ECG. Fragmented narrow QRS (various morphologies of RSR' pattern without bundle branch block) in coronary artery disease and myocardial infarction is associated with old scar and increased risk of cardiac events [1]. Fragmented wide QRS is a moderately sensitive and highly specific sign for myocardial scar in patients with known or suspected coronary artery disease [2]. Similar studies in patients undergoing nuclear stress test revealed that fQRS is associated with old scar [3]. Interestingly, this image illustrates that QRS fragmentation in almost all 12 ECG leads and is good marker of extensive myocardial scar. Fragmentation of QRS with intra-ventricular conduction delays reduced due to simultaneous left lateral ventricular pacing.

## Figures and Tables

**Figure 1B:**